# Thrombotic risk of platinum combination chemotherapy with and without immune checkpoint inhibitors for advanced non-small cell lung cancer: a nationwide inpatient database study

**DOI:** 10.1007/s00262-023-03508-1

**Published:** 2023-08-04

**Authors:** Chikako Iwai, Taisuke Jo, Takaaki Konishi, Asahi Fujita, Nobuaki Michihata, Hiroki Matsui, Kiyohide Fushimi, Hideo Yasunaga

**Affiliations:** 1 Department of Clinical Epidemiology and Health Economics, School of Public Health, The University of Tokyo, 7-3-1 Hongo, Bunkyo-Ku, Tokyo, 113-0033 Japan; 2 Department of Health Services Research, Graduate School of Medicine, The University of Tokyo, Tokyo, Japan; 3 Department of Respiratory Medicine, Graduate School of Medicine, The University of Tokyo, Tokyo, Japan; 4 Department of Ophthalmology, Graduate School of Medicine, The University of Tokyo, Tokyo, Japan; 5 Department of Health Policy and Informatics, Tokyo Medical and Dental University Graduate School, Tokyo, Japan

**Keywords:** Arterial thromboembolism, Immune checkpoint inhibitors, Lung cancer, Platinum combination chemotherapy, Venous thromboembolism

## Abstract

**Objectives:**

To determine the associated thromboembolism risk with adding immune checkpoint inhibitors (ICI) to platinum combination chemotherapy compared with platinum combination chemotherapy alone in patients with advanced non-small cell lung cancer.

**Materials and methods:**

This study identified 75,807 patients with advanced non-small cell lung cancer from the Japanese Diagnosis Procedure Combination database who started platinum combination chemotherapy between July 2010 and March 2021. The incidence of venous thromboembolism (VTE), arterial thromboembolism (ATE), and all-cause mortality within 6 months after commencing platinum combination chemotherapy was compared between patients receiving chemotherapy with ICI (ICI group, n = 7,177) and without ICI (non-ICI group, n = 37,903). Survival time analysis was performed using the overlap weighting method with propensity scores to adjust for background factors. The subdistribution hazard ratio for developing thromboembolism was calculated using the Fine-Gray model with death as a competing risk. The hazard ratio for all-cause mortality was also calculated using the Cox proportional hazards model.

**Results:**

Overall, VTE and ATE occurred in 761 (1.0%) and 389 (0.51%) patients, respectively; mortality was 11.7%. Propensity score overlap weighting demonstrated that the subdistribution hazard ratio (95% confidence interval) for VTE and ATE in the ICI group was 1.27 (1.01–1.60) and 0.96 (0.67–1.36), respectively, compared with the non-ICI group. The mortality hazard ratio in the ICI group was 0.68 (0.62–0.74).

**Conclusion:**

The addition of ICI to platinum combination therapy was associated with a higher risk of VTE compared with platinum combination therapy alone, while the risk of ATE might be comparable.

**Supplementary Information:**

The online version contains supplementary material available at 10.1007/s00262-023-03508-1.

## Introduction

Cancer patients are at increased risk for venous thromboembolism (VTE) and arterial thromboembolism (ATE) [1, 2]. The risk of cancer-related thrombosis is multifactorial, and many risk factors have been reported [3]. Among them, anticancer agents such as platinum and angiogenesis inhibitors have been found to increase the risk of VTE and ATE [3–5].

There have been an increasing number of reports regarding the risk of thromboembolism with immune checkpoint inhibitors (ICIs) [6–10] because ICIs have revolutionized the treatment of malignancies (e.g., malignant melanoma, lung cancer, and renal cell carcinoma). ICIs could increase the levels of inflammatory cytokines [11] and enhance prothrombotic conditions by activating coagulation and impairing fibrinolysis [12–14]. A cohort study (n = 2,299) using data from a U.S. database demonstrated the cumulative 6-month VTE incidence following first-line treatment for non-small cell lung cancer in patients treated with ICIs alone, chemotherapy alone, and ICI plus chemotherapy was 8.1%, 10.9%, and 12.8%, respectively [15]. Regarding the risk of developing ATE, a previous matched-pair cohort study (n = 5,684) reported a threefold higher risk of atherosclerotic cardiovascular events after initiation of ICI therapy [16]. However, none of these studies have examined the risk of thromboembolism with ICIs compared with conventional chemotherapy [17] despite the fact that additional administration of ICIs with platinum-based therapy for lung cancer is becoming common.

This study aimed to determine the risk of thromboembolism associated with adding ICIs to platinum combination chemotherapy compared with platinum combination chemotherapy alone in patients with advanced non-small cell lung cancer.

## Materials and methods

### Data source

This nationwide retrospective cohort study used the Japanese Diagnosis Procedure Combination database. The database includes discharge abstracts and administrative claims data for approximately 8,000,000 inpatient admissions from more than 1,200 hospitals throughout Japan. It covers about half of all patients admitted to acute care hospitals in Japan [18, 19]. All 82 academic hospitals are required to participate in the database, whereas the participation of community hospitals is voluntary.

The database contains the following information: unique hospital identifiers; patient age and sex; smoking history (including both current and former smoking status) at admission; body mass index (BMI) at admission; activities of daily living (ADL) at admission; dates of admission and discharge; length of hospital stay; in-hospital mortality; cancer stage; blood transfusions and medications; and interventional/surgical procedures indexed by original Japanese codes. Diagnoses, comorbidities, and complications are recorded using the International Classification of Diseases, Tenth Revision (ICD-10) codes, and Japanese text data. The database includes no laboratory data. A previous validation study showed good sensitivity and specificity for the diagnoses and procedures recorded in this database [18].

The need for informed consent for this study was waived because the patient database was anonymized. The study was approved by the Institutional Review Board of the University of Tokyo (Approval number: 3501– (5), May 19, 2021).

### Patient selection

Patients hospitalized for the first administration of platinum combination therapy for advanced non-small cell lung cancer between July 1, 2010, and March 31, 2021, were identified. Non-small cell lung cancer was identified using the ICD-10 code of C34 and Japanese text data. Platinum combination therapy was defined as the following regimens, including cisplatin (CDDP) or carboplatin (CBDCA): (a) CDDP plus pemetrexed (PEM), (b) CBDCA plus PEM, (c) CBDCA plus nab-paclitaxel (nabPTX), (d) CBDCA plus paclitaxel (PTX), (e) CBDCA plus PTX plus bevacizumab (BEV), and (f) above-stated regimens plus ICI (pembrolizumab or atezolizumab). The ICIs used in each regimen were described.

Eligible patients were divided into the ICI and non-ICI groups. The ICI group included patients administered platinum combination regimens plus an ICI (pembrolizumab or atezolizumab), and the non-ICI group included those without ICI therapy. The following patients were excluded: (i) those with pulmonary sarcoma, pediatric pleuropulmonary blastoma, or pulmonary malignant melanoma; (ii) those aged less than 18 years; (iii) those who started chemotherapy after October 1, 2020 (because of the observation period being less than 6 months); (iv) those treated with multiple regimens in the same hospitalization; (v) those who received anticancer agents of the regimen on separate days; (vi) those who received multiple cycles of platinum combination regimens in the same hospitalization; (vii) those who were administered anticoagulants (direct oral anticoagulants [dabigatran, rivaroxaban, apixaban, and edoxaban] and warfarin) within the past 1 year; and (viii) those who had experienced VTE or ATE within the past 1 year.

### Outcomes

The primary outcomes were VTE and ATE, requiring hospitalization within 6 months after the start of platinum combination chemotherapy. VTE included deep vein thrombosis (ICD-10 codes: I80.1, I80.2, I80.3, I80.8, I80.9, and I82.8) and pulmonary embolism (I26.0 and I26.9) [20]. According to previous studies, ATE was defined as ischemic heart disease (I20.0, I20.1, I20.8, I20.9, I21.0, I21.1, I21.2, I21.3, I21.4, and I21.9), ischemic brain disease (I63 and G45.9), and peripheral arterial occlusion (I74.0, I74.1, I74.2, I74.3, I74.4, I74.5, I74.8, and I74.9) [1, 8]. VTE or ATE onset was defined as the date when the patient received direct oral anticoagulants or warfarin for a VTE or ATE diagnosis. The secondary outcome was all-cause in-hospital death within 6 months after the start of platinum combination chemotherapy.

### Covariates

Covariates were age, sex, BMI, weight, smoking status (nonsmoker, current/past smoker, missing), ADL at admission, combined small cell lung carcinoma, comorbidities, clinical cancer stage (III or IV), treatments before admission (molecular-targeted medications, dialysis, radiotherapy, and surgery within 6 months prior to the index hospitalization), pretreatment medications and procedures during the index hospitalization, baseline chemotherapy, days from admission to initiation of chemotherapy, type of hospital (academic hospital or non-academic hospital), and hospital volume.

Age was categorized into six groups: 18–39, 40–49, 50–59, 60–69, 70–79, and 80 or more years. BMI was categorized using the World Health Organization classifications: less than 18.5 kg/m^2^ (underweight), 18.5–24.9 kg/m^2^ (normal weight), 25.0–29.9 kg/m^2^ (overweight), and more than or equal to 30.0 kg/m^2^ (obese and severely obese). ADL at admission was assessed using the Barthel index and categorized into three groups: less than or equal to 40, 45–80, and 85–100 [21, 22]. Comorbidities were investigated using the ICD-10 codes (Supplemental Table 1) and assessed using the Charlson comorbidity index [23]. The index was categorized into three groups: less than or equal to 2, 3–4, and more than or equal to 5. Pretreatment medications included antihypertensives, antiplatelet drugs, antipsychotics, corticosteroids, estrogen preparations, non-steroidal anti-inflammatory drugs, proton pump inhibitors/potassium-competitive acid, and statins. Pretreatment procedures included central venous catheterization, radiotherapy, surgery, and transfusion of red cell concentrate. A previous study reported that CDDP-based regimens were associated with a higher risk of VTE than CBDCA-based regimens in patients with lung cancer [24]; therefore, to account for differences in the risk of thrombosis, we adjusted for the baseline chemotherapy regimens in the current analysis. Hospital volume was defined as the annual number of eligible patients at each hospital and categorized into tertiles with approximately equal numbers of patients in each group.

**Table 1:** Patient background before and after overlap weighting

	Before overlap weighting						After overlap weighting				
	ICI n = 7,177		non-ICI n = 68,630		ASD^*^(%)		ICI n = 37,903		non-ICI n = 37,903		ASD* (%)
Age, years											
< 40	60	(0.8)	849	(1.2)	4.0		335	(0.9)	335	(0.9)	0.0
40–49	388	(5.4)	3,246	(4.7)	3.1		2,029	(5.4)	2,029	(5.4)	0.0
50–59	1,090	(15)	10,191	(15)	0.9		5,746	(15)	5,746	(15)	0.0
60–69	2,877	(40)	29,339	(43)	5.4		15,305	(40)	15,305	(40)	0.0
70–79	2,636	(37)	23,167	(34)	6.2		13,781	(36)	13,781	(36)	0.0
≥ 80	126	(1.8)	1,838	(2.7)	6.3		707	(1.9)	707	(1.9)	0.0
Sex (male)	5,600	(78)	49,867	(73)	12.5		29,310	(77)	29,310	(77)	0.0
Body mass index, kg/m^2^											
< 18.5	1,137	(16)	9,858	(14)	4.1		5,964	(16)	5,964	(16)	0.0
18.5–24.9	4,786	(67)	46,119	(67)	1.1		25,295	(67)	25,295	(67)	0.0
25.0–29.9	1,083	(15)	10,804	(16)	1.8		5,732	(15)	5,732	(15)	0.0
≥ 30.0	129	(1.8)	1,276	(1.9)	0.5		676	(1.8)	676	(1.8)	0.0
Missing data	42	(0.6)	573	(0.8)	3.0		236	(0.6)	236	(0.6)	0.0
Smoking											
Nonsmoker	1,469	(20)	18,923	(28)	16.7		8,090	(21)	8,090	(21)	0.0
Current/past smoker	5,155	(72)	44,542	(65)	14.9		26,880	(71)	26,880	(71)	0.0
Missing data	553	(7.7)	5,165	(7.5)	0.7		2,933	(7.7)	2,933	(7.7)	0.0
Barthel index at admission											
≤ 40	79	(1.1)	734	(1.1)	0.3		416	(1.1)	416	(1.1)	0.0
45–80	270	(3.8)	2,519	(3.7)	0.5		1,430	(3.8)	1,430	(3.8)	0.0
85–100	6,565	(91)	62,884	(92)	0.6		34,670	(91)	34,670	(91)	0.0
Missing data	263	(3.7)	2,493	(3.6)	0.2		1,387	(3.7)	1,387	(3.7)	0.0
Combined SCLC	18	(0.3)	768	(1.1)	10.5		106	(0.3)	106	(0.3)	0.0
Comorbidities											
Atrial fibrillation	54	(0.8)	807	(1.2)	4.3		308	(0.8)	308	(0.8)	0.0
Autoimmune disease	41	(0.6)	1,173	(1.7)	10.7		246	(0.6)	246	(0.6)	0.0
Chronic kidney disease	30	(0.4)	397	(0.6)	2.3		172	(0.5)	172	(0.5)	0.0
Chronic pulmonary disease	791	(11)	10,469	(15)	12.6		4,418	(12)	4,418	(12)	0.0
Congestive heart failure	97	(1.4)	1,233	(1.8)	3.6		544	(1.4)	544	(1.4)	0.0
Chronic interstitial lung disease	93	(1.3)	3,649	(5.3)	22.6		563	(1.5)	563	(1.5)	0.0
Dementia	18	(0.3)	154	(0.2)	0.5		91	(0.2)	91	(0.2)	0.0
Diabetes with chronic complication	80	(1.1)	1,007	(1.5)	3.1		438	(1.2)	438	(1.2)	0.0
Diabetes without chronic complication	879	(12)	9,393	(14)	4.3		4,776	(13)	4,776	(13)	0.0
Dyslipidemia	431	(6.0)	5,170	(7.5)	6.1		2,390	(6.3)	2,390	(6.3)	0.0
Hemiplegia or paraplegia	15	(0.2)	160	(0.2)	0.5		80	(0.2)	80	(0.2)	0.0
Hypertension	1,079	(15)	13,882	(20)	13.7		6,019	(16)	6,019	(16)	0.0
Intracerebral bleeding	6	(0.1)	54	(0.1)	0.2		30	(0.1)	30	(0.1)	0.0
Mild liver disease	169	(2.4)	2,036	(3.0)	3.8		943	(2.5)	943	(2.5)	0.0
Moderate or severe liver disease	3	(0.0)	19	(0.0)	0.8		15	(0.0)	15	(0.0)	0.0
Other interstitial lung diseases	8	(0.1)	342	(0.5)	7.0		49	(0.1)	49	(0.1)	0.0
Peptic ulcer disease	198	(2.8)	4,860	(7.1)	20.1		1,160	(3.1)	1,160	(3.1)	0.0
Psychoses	48	(0.7)	690	(1.0)	3.7		271	(0.7)	271	(0.7)	0.0
Charlson comorbidity index											
≤ 2	4,533	(63)	40,965	(60)	7.1		23,552	(62)	23,552	(62)	0.0
3–4	78	(1.1)	1,300	(1.9)	6.7		442	(1.2)	442	(1.2)	0.0
≥ 5	2,566	(36)	26,365	(38)	5.5		13,909	(37)	13,909	(37)	0.0

Data are presented as n (%) or mean (standard deviation). ASD, absolute standardized difference; ICI, immune checkpoint inhibitor; SCLC, small cell lung carcinoma

^*^An ASD of < 10% denotes a negligible difference between the two groups

### Statistical analysis

We conducted propensity-score overlap weighting to control for potential confounding factors. The overlap weighting analysis balanced the two groups by minimizing the asymptotic variance of the nonparametric estimates of the weighted average treatment effect within a class of weights [25–30]. The two groups were balanced by overlap weighting based on propensity scores estimated from the logistic regression analysis using the above covariates as potential confounders. The weights were constructed as the probability of each patient receiving the opposite treatment. Standardized differences were calculated to assess the balance of covariates between the two groups; an absolute value of the standardized difference being 10% or more indicates that the covariate is significantly unbalanced [31].

A Cox proportional hazards regression model with adjustment for within-hospital clustering was used for the two weighted groups. Generalized estimating equations were used to calculate the hazard ratio (HR) with 95% confidence intervals (CIs) for the association with outcomes. Cumulative incidence functions, adjusted for competing risks, were used to account for VTE or ATE onset. A competing risk is an event that prevents the occurrence of the primary events of interest [32]. This study's competing risk was in-hospital death from causes other than VTE or ATE. For the analysis of VTE or ATE incidence, the Fine-Gray model was used to calculate subdistribution HRs (SHRs). The Fine-Gray model is recommended for incidence probability and predictive modeling in the presence of competing risks [32, 33].

We performed stratified analyses according to age (< 70 and ≥ 70 years [34] and < 75 and ≥ 75 years [35, 36]) and ICI drugs (pembrolizumab and atezolizumab). Furthermore, we divided propensity score-weighted patients according to age and ICI drugs. Additionally, two sensitivity analyses were performed to assess the robustness of our findings. First, those who received bevacizumab-containing regimens were excluded because bevacizumab is known to be associated with thrombosis [4]. Second, the instrumental variables method, which allows for the adjustment of residual unmeasured confounders, was used [37]. “The proportion of ICIs used in the number of chemotherapy administrations in each hospital” was used as the instrumental variable. This is because it can meet the general criteria for instrumental variables as follows: (i) it was not associated with patient background characteristics, (ii) it was highly associated with treatment choice, and (iii) it did not affect patient outcomes except through the treatment [38–40]. F-statistics were tested to confirm the validity of the instrumental variables, and an F-statistic less than 10 was considered an invalid instrumental variable [40]. A competing risk analysis, like the main analysis, was performed for VTE and ATE incidence.

All hypothesis tests employed a two-sided statistical significance level of 0.05, and all statistical analyses were performed using Stata/SE 17.0 statistical software (StataCorp, College Station, TX, USA).

## Results

**Fig. 1:**
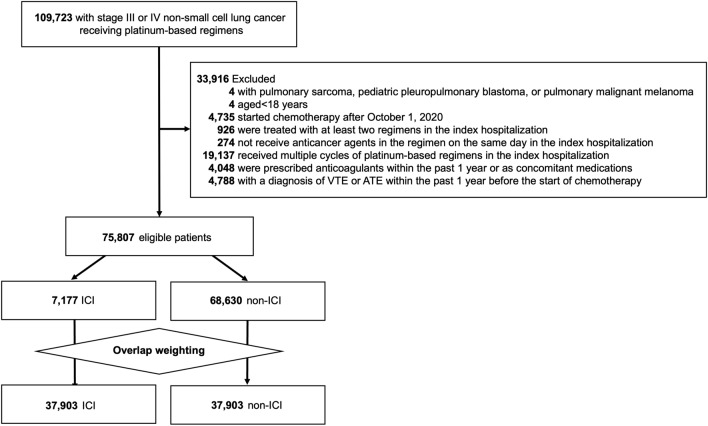
Flow diagram, VTE, venous thromboembolism; ATE, arterial thromboembolism; ICI, immune checkpoint inhibitor

This study identified 109,723 patients with stage III or IV lung cancer who started treatment with a platinum-based regimen from July 1, 2010, to March 31, 2021. Then 33,916 patients were excluded based on the exclusion criteria shown in Fig. 1. Of the 75,807 eligible patients, the ICI and non-ICI groups comprised 7,177 and 68,630 patients, respectively.

Table 1 shows the baseline characteristics before and after propensity score overlap weighting for patients treated with and without ICI. Before adjustment for overlap weighting, the ICI group was significantly more likely to be male, in teaching hospitals, and current or past smokers.

Table 2 shows comorbidity and baseline medication information, including baseline chemotherapy regimen. Before adjustment for overlap weighting, the ICI group was significantly more likely to have stage IV cancer and have shorter intervals from admission to the start of chemotherapy than the non-ICI group. Regarding chemotherapy regimens, the ICI group was significantly more likely to use CBDCA plus PEM, CBDCA plus nabPTX, and CBDCA plus PTX plus BEV, and significantly less likely to use CDDP plus PEM and CBDCA plus PTX. Supplemental Table 2 shows the details of which ICI was used in each regimen. The total numbers of patients treated with pembrolizumab and atezolizumab were 6,186 and 991 before overlap weighting, and 32,976 and 4,957 after overlap weighting, respectively. The ICI group was significantly less likely to have hypertension, chronic interstitial pneumonia, chronic lung disease, autoimmune disease, and peptic ulcer disease. After adjustment for propensity score overlap weighting, each group comprised 37,903 patients, and the backgrounds were completely balanced (that is, all the absolute standardized differences were zero).

**Table 2:** Treatment background before and after overlap weighting

	Before overlap weighting						After overlap weighting				
	ICI n = 7,177		non-ICI n = 68,630		ASD^†^ (%)		ICI n = 37,903		non-ICI n = 37,903		ASD^†^ (%)
Clinical cancer stage											
III	1,102	(15)	16,727	(24)	22.7		6,166	(16)	6,166	(16)	0.0
IV	6,075	(85)	51,903	(76)	22.7		31,737	(84)	31,737	(84)	0.0
Treatments before admission											
Administration of BEV or ramucirumab	5	(0.1)	119	(0.2)	3.0		30	(0.1)	30	(0.1)	0.0
Dialysis	4	(0.06)	43	(0.1)	0.3		23	(0.1)	23	(0.1)	0.0
Prescription of EGFR-TKI	87	(1.2)	1,962	(2.9)	11.7		505	(1.3)	505	(1.3)	0.0
Radiotherapy	379	(5.3)	2,639	(3.8)	6.9		1,911	(5.0)	1,911	(5.0)	0.0
Surgery	787	(11)	7,189	(10)	1.6		4,101	(11)	4,101	(11)	0.0
Pretreatment medications											
Antihypertensives^*^	2,631	(37)	18,971	(28)	19.4		13,277	(35)	13,277	(35)	0.0
Antiplatelet drugs	622	(8.7)	4,226	(6.2)	9.6		3,097	(8.2)	3,097	(8.2)	0.0
Antipsychotics	1,954	(27)	13,878	(20)	16.5		9,938	(26)	9,938	(26)	0.0
Corticosteroids	3,380	(47)	29,528	(43)	8.2		17,727	(47)	17,727	(47)	0.0
Estrogen preparations	10	(0.1)	82	(0.1)	0.6		50	(0.1)	50	(0.1)	0.0
NSAIDs	2,948	(41)	27,477	(40)	2.1		15,470	(41)	15,470	(41)	0.0
Osteoporosis drugs	28	(0.4)	158	(0.2)	2.9		133	(0.4)	133	(0.4)	0.0
PPI/ Potassium-competitive acid	3,315	(46)	26,790	(39)	14.5		17,104	(45)	17,104	(45)	0.0
Statins	1,077	(15)	6,779	(9.9)	15.6		5,328	(14)	5,328	(14)	0.0
Pretreatment procedures											
Central venous catheterization	28	(0.4)	266	(0.4)	0.04		142	(0.4)	142	(0.4)	0.0
Radiotherapy	471	(6.6)	4,049	(5.9)	2.7		2,463	(6.5)	2,463	(6.5)	0.0
Surgery	60	(0.8)	676	(1.0)	1.6		320	(0.8)	320	(0.8)	0.0
Transfusion of red cell concentrate	66	(0.9)	546	(0.8)	1.3		338	(0.9)	338	(0.9)	0.0
Baseline chemotherapy											
CDDP plus PEM	894	(12)	14,412	(21)	23.0		5,159	(14)	5,159	(14)	0.0
CBDCA plus PEM	3,050	(42)	24,014	(35)	15.5		16,243	(43)	16,243	(43)	0.0
CBDCA plus nabPTX	2,219	(31)	12,001	(17)	31.8		11,119	(29)	11,119	(29)	0.0
CBDCA plus PTX	296	(4.1)	14,466	(21)	52.8		1,798	(4.7)	1,798	(4.7)	0.0
CBDCA plus PTX plus BEV	718	(10)	3,737	(5.4)	17.1		3,584	(9.5)	3,584	(9.5)	0.0
Days to start chemotherapy, days	4.0	(6.8)	4.7	(7.3)	9.1		4.1	(7.0)	4.1	(6.1)	0.0
Academic hospital	1,657	(23)	12,358	(18)	12.6		8,384	(22)	8,384	(22)	0.0
Hospital volume											
< 14	2,522	(35)	22,651	(33)	4.5		13,274	(35)	13,274	(35)	0.0
14–24	2,578	(36)	22,717	(33)	5.9		13,440	(35)	13,440	(35)	0.0
≥ 24	2,077	(29)	23,262	(34)	10.7		11,189	(30)	11,189	(30)	0.0

Data are presented as n (%) or mean (standard deviation)

ASD, absolute standardized difference; ICI, immune checkpoint inhibitor CDDP, cisplatin; CBDCA, carboplatin; PEM, pemetrexed; nabPTX nab-paclitaxel; PTX, paclitaxel; BEV, bevacizumab; ACE-I, angiotensin-converting enzyme inhibitor; ARB, angiotensin II receptor blocker; CYP, cytochrome P450; EGFR-TKI, epidermal growth factor receptor tyrosine kinase inhibitor; NSAIDs, non-steroidal anti-inflammatory drugs; PPI, Proton pump inhibitors

^*^Antihypertensive drugs included angiotensin-converting enzyme inhibitors, angiotensin II receptor blockers, β-blockers, and Ca-blockers

^†^An ASD of < 10% denotes a negligible difference between the two groups

**Fig. 2:**
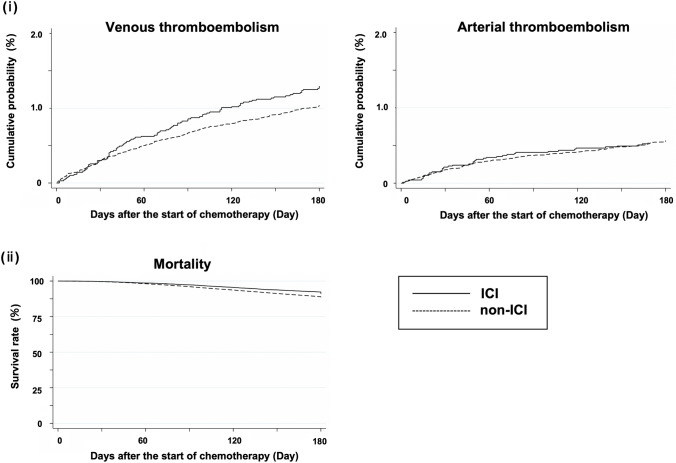
Incidence of outcomes in hospitalizations within 6 months after the start of platinum combination chemotherapy **i** Cumulative probability for venous and arterial thromboembolism events, **ii** Survival curves for mortality ICI, immune checkpoint inhibitor

Table 3 shows the proportion of VTE and ATE requiring anticoagulation within 6 months after the start of platinum combination chemotherapy and the proportion of all-cause in-hospital death within 6 months of commencing platinum combination chemotherapy. Supplementary Table 3 shows the number of events of VTE (deep vein thrombosis and pulmonary embolism) and ATE (ischemic heart disease, ischemic brain disease, and peripheral arterial embolism). Cumulative probabilities for VTE and ATE and survival curves are shown in Fig. 2. Before weighting, VTE incidence was 1.3% and 0.97%, ATE incidence was 0.52% and 0.51%, and the mortality was 8.7% and 12% in the ICI and non-ICI groups, respectively.

**Table 3:** Incidence of outcomes before and after overlap weighting

	Before overlap weighting					After overlap weighting			
	ICI n = 7,177		non-ICI n = 68,630			ICI n = 37,903		non-ICI n = 37,903	
Venous thromboembolism	96	(1.3)	665	(0.97)		505	(1.3)	389	(1.0)
Arterial thromboembolism	38	(0.52)	351	(0.51)		204	(0.54)	221	(0.58)
All-cause in-hospital death	626	(8.7)	8,211	(12)		3,297	(8.7)	4,709	(12)

ICI, immune checkpoint inhibitor

All outcomes were events requiring hospitalization within 6 months after the start of platinum combination chemotherapy. Data are shown in n (%)

**Fig. 3:**
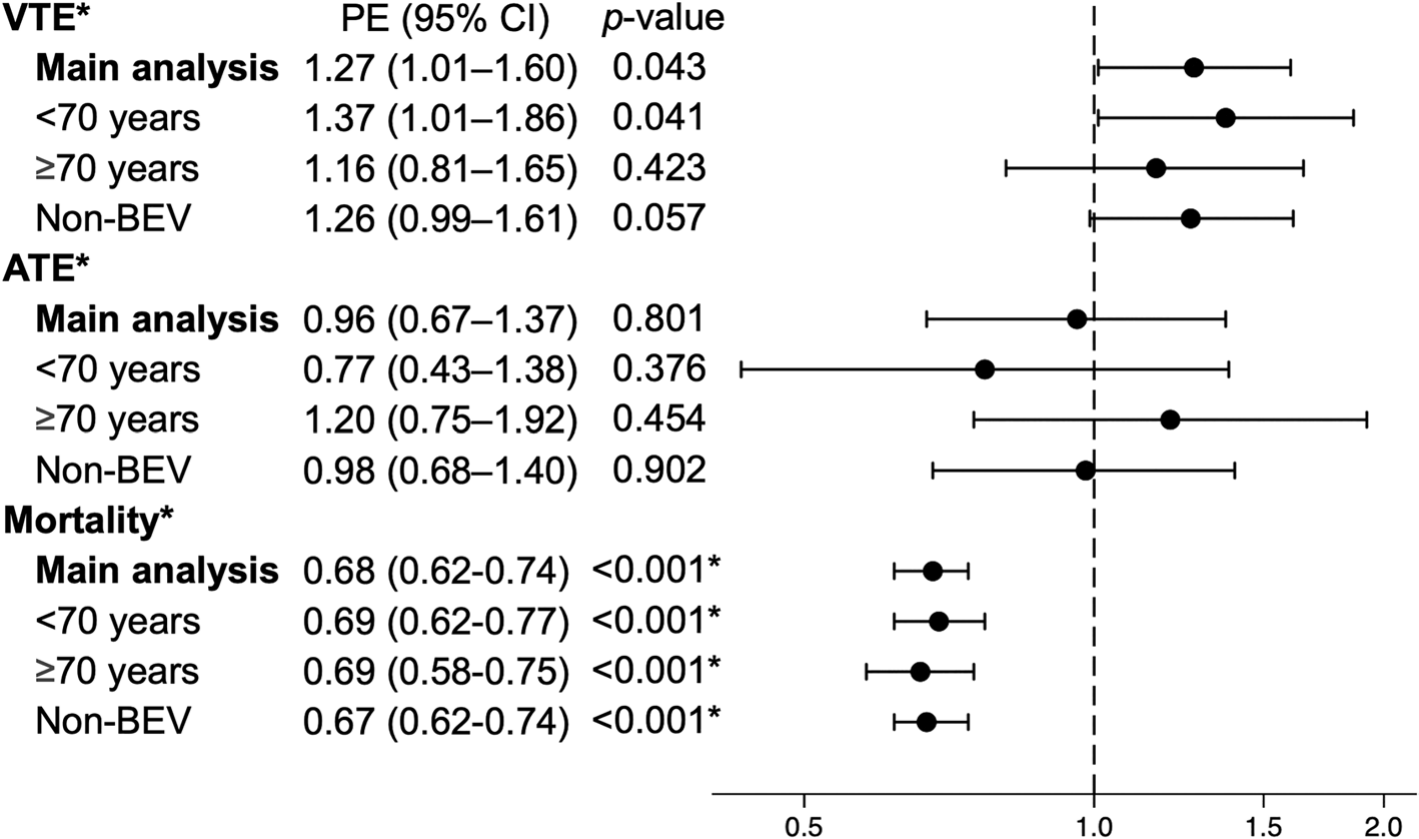
Point estimates for outcomes within 6 months after the start of platinum combination chemotherapy VTE, venous thromboembolism; ATE, arterial thromboembolism; BEV, bevacizumab; PE, point estimate; CI, confidence interval Point estimates for VTE and ATE show subdistribution hazard ratios in the competing risk analysis. Point estimates for mortality show the hazard ratio. *Mortality represents all-cause in-hospital death. All outcomes were events requiring hospitalization within 6 months after the start of platinum combination chemotherapy

Figure 3 shows the SHR and HR of the ICI group versus the non-ICI group. The SHR for the main analysis was significantly higher in the ICI group for VTE in hospitalizations within 6 months after the start of platinum combination chemotherapy at 1.27 (95% CI, 1.01–1.60) and not significantly different for ATE at 0.96 (0.67–1.36). All-cause in-hospital mortality was significantly lower, with a HR of 0.68 (0.62–0.74). Stratified and sensitivity analyses showed similar trends as the main analysis (Fig. 3). Patients less than 70 years old were more likely to develop VTE than those who were more than 70 years old; whereas, risks for VTE and ATE did not significantly differ between the ICI and non-ICI groups in the analysis stratified by age less than 75 and more than 75 years (Supplemental Table 4). The results of the stratified analyses for pembrolizumab were similar to those of the main analyses (Supplemental Table 5). The SHRs for VTE and ATE at instrumental variables method were 1.16 (0.29–4.67) and 0.10 (0.01–1.16), respectively. The HR for mortality was 0.62 (0.38–1.02), and the F-statistic was 656.

## Discussion

This study compared the thrombosis risk of platinum combination therapy with and without ICI in patients with advanced non-small cell lung cancer using a nationwide Japanese inpatient database. The study showed that the ICI group was associated with an increased risk of in-hospital VTE and a decreased risk of all-cause in-hospital mortality within 6 months of commencing platinum combination chemotherapy compared with the non-ICI group. In addition, the results of the stratified analyses by age (< 70 and ≥ 70 years) and sensitivity analyses corresponded with the main analyses results.

In the unadjusted cohort, the ICI group was more likely to be hospitalized in teaching hospitals than the non-ICI group. Academic hospitals with multidisciplinary and multispecialty teams would have allowed ICI administration because ICI can cause various systemic immune-related side effects [41, 42]. In addition, since ICI administration may be contraindicated in patients with chronic pulmonary (such as interstitial lung disease) and autoimmune diseases, these diseases were less common in the ICI group than in the non-ICI group. These differences were well-adjusted in the current overlap weighting analysis.

A previous study compared the incidence between 2 years before ICI administration and 2 years after administration and reported a more than four-fold increase in VTE incidence after ICI administration [7]. In addition, a history of hypertension and age less than 65 years were also identified as predictive factors for VTE. In the present study, the SHR for VTE was significantly increased in the ICI group compared with the non-ICI group, suggesting that ICI administration may be a risk factor for VTE. Compared with previous studies, the rate of increase was low. This may be because the comparison group in this study focused on platinum combination chemotherapy. Our stratified analysis also suggested that the risk of VTE may be higher in younger patients (< 70 years of age), similar to a previous report [7]. Additionally, the stratified analysis by ICI drugs revealed that regimens with pembrolizumab were associated with a higher risk of VTE compared with regimens without pembrolizumab. Previous studies reported that the risk of VTE was equivalent among different ICIs [8]. The present study observed no significant difference in the risk of developing ATE between the ICI and non-ICI groups. A past matched-pair cohort study had an average BMI or more than 27 kg/m^2^ [16], but the current study had a different population with BMIs less than 25 kg/m^2^in more than 80% of participants; this difference might have resulted in the low ATE incidence regardless of ICI administration in the current cohort. Additionally, the difference in chemotherapy combined with ICI in the present study may have affected ATE incidence. Platinum chemotherapy requires high-volume infusion and special attention to cardiac function [43]. Thus, clinicians would have selected patients with good cardiac function in the current population treated with platinum chemotherapy.

All-cause mortality within 6 months was significantly lower in the ICI group than in the non-ICI group (Fig. 2). The reduced mortality in the present study is similar to previous randomized controlled trials (e.g., the HR for mortality was 0.71 in KEYNOTE-407) [44–47]. These trials demonstrated long-term survival (approximately 17–22 months) in patients treated with ICI, whereas the current study revealed that ICI use was associated with a better prognosis even in a shorter period.

The strength of this study lies in the fact that it was conducted in a real-world clinical setting and compared several advanced lung cancer patients to determine whether the addition of ICI increases the risk of thromboembolism, using a platinum-based chemotherapy arm as a control: we showed that the risk of venous thrombosis is approximately 1.3-fold higher with the addition of ICIs. Furthermore, instrumental variable methods were used to adjust for unmeasured confounding, and the results showed a similar trend, indicating that the results are robust. We believe that the current study may provide important information in the decision-making process for lung cancer treatment. However, this study had some limitations. First, outpatient treatment data were unavailable. In Japan, most platinum combination chemotherapies are initiated in an inpatient setting [24]. Therefore, the first administration of the target regimen could be identified using inpatient data only. Second, the unavailability of echocardiographic and electrocardiographic results and information on programmed cell death-ligand 1 antibodies could have led to a bias in the treatment choice to administer ICI. Furthermore, information on histological types (squamous cell carcinoma, adenocarcinoma) and oncogenic gene mutations could not be obtained from the database [48]. In lung and esophageal cancer, the risk of VTE has been reported to be higher for adenocarcinoma than for squamous cell carcinoma [49, 50]. Oncogenic gene mutations, especially ALK fusion gene mutations, are known risk factors for thrombosis [51]. However, the instrumental variable method can adjust for such unmeasured confounders and would support the results of the main analyses. Third, information on outcomes outside the hospital (e.g., thromboembolisms treated in other hospitals and death at home) could not be obtained. Mild venous thrombosis without inpatient treatment may also have been missed. Hence, there may be an underestimation of the outcomes. Moreover, angina pectoris treated only with antiplatelet agents was not included as an outcome. However, because the lack of information is expected to occur equally in the two groups, they are not likely to skew the results. Fourth, with the approval of direct oral anticoagulants, the detection of thrombosis in the ICI and non-ICI groups may be different. For example, in Trousseau syndrome, heparin and direct oral anticoagulants are more likely to be used and warfarin is less frequently selected. Patients with ischemic brain disease in the non-ICI group before direct oral anticoagulants approval may not have been treated with warfarin and thus may have been undetected. Finally, since patients with a history of thromboembolism and patients already taking anticoagulants were excluded, further investigation on this population is needed.

## Conclusion

Adding ICIs to platinum combination chemotherapy was associated with an increased risk of in-hospital VTE within 6 months after the start of platinum combination chemotherapy compared with platinum combination chemotherapy alone, while the risk of in-hospital ATE was similar. Therefore, clinicians should closely monitor patients for the risk of thromboembolism when using regimens with ICI added to platinum combination chemotherapy.

### Supplementary Information

Below is the link to the electronic supplementary material.

Supplementary file1 (DOCX 34 KB)

## Data Availability

The data analyzed during the current study are not publicly available due to contracts with the hospitals providing data to the database. Further inquiries on data can be directed to the corresponding author.

## References

[1] Grilz E, Königsbrügge O, Posch F (2018). Frequency, risk factors, and impact on mortality of arterial thromboembolism in patients with cancer. Haematologica.

[2] Cohen AT, Katholing A, Rietbrock S (2017). Epidemiology of first and recurrent venous thromboembolism in patients with active cancer a population-based cohort study. Thromb Haemost.

[3] Ay C, Pabinger I, Cohen AT (2017). Cancer-associated venous thromboembolism: burden, mechanisms, and management. Thromb Haemost.

[4] Nalluri SR, Chu D, Keresztes R (2008). Risk of venous thromboembolism with the angiogenesis inhibitor bevacizumab in cancer patients: a meta-analysis. JAMA.

[5] Totzeck M, Mincu RI, Rassaf T (2017). Cardiovascular adverse events in patients with cancer treated with bevacizumab: a meta-analysis of more than 20 000 patients. J Am Heart Assoc.

[6] Bar J, Markel G, Gottfried T (2019). Acute vascular events as a possibly related adverse event of immunotherapy: a single-institute retrospective study. Eur J Cancer.

[7] Gong J, Drobni ZD, Alvi RM (2021). Immune checkpoint inhibitors for cancer and venous thromboembolic events. Eur J Cancer.

[8] Moik F, Chan W-SE, Wiedemann S (2021). Incidence, risk factors, and outcomes of venous and arterial thromboembolism in immune checkpoint inhibitor therapy. Blood.

[9] Ando Y, Hayashi T, Sugimoto R (2020). Risk factors for cancer-associated thrombosis in patients undergoing treatment with immune checkpoint inhibitors. Invest New Drugs.

[10] Deschênes-Simard X, Richard C, Galland L (2021). Venous thrombotic events in patients treated with immune checkpoint inhibitors for non-small cell lung cancer: a retrospective multicentric cohort study. Thromb Res.

[11] Urwyler P, Earnshaw I, Bermudez M (2020). Mechanisms of checkpoint inhibition-induced adverse events. Clin Exp Immunol.

[12] Esmon CT (2003). Inflammation and thrombosis. J Thromb Haemost.

[13] Franco AT, Corken A, Ware J (2015). Platelets at the interface of thrombosis, inflammation, and cancer. Blood.

[14] Engelmann B, Massberg S (2013). Thrombosis as an intravascular effector of innate immunity. Nat Rev Immunol.

[15] Khorana AA, Palaia J, Rosenblatt L (2023). Venous thromboembolism incidence and risk factors associated with immune checkpoint inhibitors among patients with advanced non-small cell lung cancer. J Immunother Cancer.

[16] Drobni ZD, Alvi RM, Taron J (2020). Association between immune checkpoint inhibitors with cardiovascular events and atherosclerotic plaque. Circulation.

[17] Giustozzi M, Becattini C, Roila F (2021). Vascular events with immune checkpoint inhibitors in melanoma or non-small cell lung cancer: a systematic review and meta-analysis. Cancer Treat Rev.

[18] Yamana H, Moriwaki M, Horiguchi H (2017). Validity of diagnoses, procedures, and laboratory data in Japanese administrative data. J Epidemiol.

[19] Yasunaga H (2019). Real world data in Japan: chapter II the diagnosis procedure combination database. Ann Clin Epidemiol.

[20] Iwai C, Jo T, Konishi T (2023). Comparative safety and effectiveness of direct oral anticoagulants and warfarin during chemotherapy in cancer patients with venous thromboembolism aged 75 years or older: a nationwide inpatient database study. Gerontology.

[21] Katano S, Yano T, Ohori K (2022). Barthel index score predicts mortality in elderly heart failure–a goal of comprehensive cardiac rehabilitation. Circ J.

[22] Konishi T, Sakata A, Inokuchi H (2022). Treatments and outcomes of adult parapharyngeal and retropharyngeal abscess: 1882 cases from a Japanese nationwide database. Am J Otolaryngol.

[23] Charlson ME, Pompei P, Ales KL, MacKenzie CR (1987). A new method of classifying prognostic comorbidity in longitudinal studies: development and validation. J Chronic Dis.

[24] Mitani A, Jo T, Yasunaga H (2018). Venous thromboembolic events in patients with lung cancer treated with cisplatin-based versus carboplatin/nedaplatin-based chemotherapy. Anticancer Drugs.

[25] Desai RJ, Franklin JM (2019). Alternative approaches for confounding adjustment in observational studies using weighting based on the propensity score: a primer for practitioners. BMJ.

[26] Thomas LE, Li F, Pencina MJ (2020). Overlap weighting: a propensity score method that mimics attributes of a randomized clinical trial. JAMA.

[27] Mehta N, Kalra A, Nowacki AS (2020). Association of use of angiotensin-converting enzyme inhibitors and angiotensin II receptor blockers with testing positive for coronavirus disease 2019 (COVID-19). JAMA Cardiol.

[28] Xian Y, Xu H, O’Brien EC (2019). Clinical effectiveness of direct oral anticoagulants vs warfarin in older patients with atrial fibrillation and ischemic stroke: findings from the patient-centered research into outcomes stroke patients prefer and effectiveness research (PROSPER) study. JAMA Neurol.

[29] Li F, Morgan KL, Zaslavsky AM (2018). Balancing covariates via propensity score weighting. J Am Stat Assoc.

[30] Li F, Thomas LE, Li F (2019). Addressing extreme propensity scores via the overlap weights. Am J Epidemiol.

[31] Austin PC (2009). Balance diagnostics for comparing the distribution of baseline covariates between treatment groups in propensity-score matched samples. Stat Med.

[32] Noordzij M, Leffondré K, van Stralen KJ (2013). When do we need competing risks methods for survival analysis in nephrology?. Nephrol Dial Transplant.

[33] Austin PC, Lee DS, Fine JP (2016). Introduction to the analysis of survival data in the presence of competing risks. Circulation.

[34] Froesch P, Martucci F, Györik S (2014). Management of non-small cell lung cancer in the elderly. Eur J Intern Med.

[35] Fujimoto D, Miura S, Yoshimura K (2022). A real-world study on the effectiveness and safety of pembrolizumab plus chemotherapy for nonsquamous NSCLC. JTO Clin Res Rep.

[36] Morimoto K, Yamada T, Yokoi T (2021). Clinical impact of pembrolizumab combined with chemotherapy in elderly patients with advanced non-small-cell lung cancer. Lung Cancer.

[37] Newhouse JP, McClellan M (1998). Econometrics in outcomes research: the use of instrumental variables. Annu Rev Public Health.

[38] Brookhart MA, Rassen JA, Schneeweiss S (2010). Instrumental variable methods in comparative safety and effectiveness research. Pharmacoepidemiol Drug Saf.

[39] Greenland S (2000). An introduction to instrumental variables for epidemiologists. Int J Epidemiol.

[40] Baiocchi M, Cheng J, Small DS (2014). Tutorial in biostatistics: instrumental variable methods for causal inference*. Stat Med.

[41] Champiat S, Lambotte O, Barreau E (2016). Management of immune checkpoint blockade dysimmune toxicities: a collaborative position paper. Ann Oncol.

[42] Brahmer JR, Lacchetti C, Schneider BJ (2018). Management of immune-related adverse events in patients treated with immune checkpoint inhibitor therapy: American society of clinical oncology clinical practice guideline. J Clin Oncol.

[43] Dugbartey GJ, Peppone LJ, de Graaf IAM (2016). An integrative view of cisplatin-induced renal and cardiac toxicities: molecular mechanisms, current treatment challenges and potential protective measures. Toxicology.

[44] Paz-Ares L, Vicente D, Tafreshi A (2020). A randomized, placebo-controlled trial of pembrolizumab plus chemotherapy in patients with metastatic squamous NSCLC: protocol-specified final analysis of KEYNOTE-407. J Thorac Oncol.

[45] West H, McCleod M, Hussein M (2019). Atezolizumab in combination with carboplatin plus nab-paclitaxel chemotherapy compared with chemotherapy alone as first-line treatment for metastatic non-squamous non-small-cell lung cancer (IMpower130): a multicentre, randomised, open-label, phase 3 trial. Lancet Oncol.

[46] Nishio M, Barlesi F, West H (2021). Atezolizumab plus chemotherapy for first-line treatment of nonsquamous NSCLC: results from the randomized phase 3 IMpower132 Trial. J Thorac Oncol.

[47] Gadgeel S, Rodríguez-Abreu D, Speranza G (2020). Updated analysis from KEYNOTE-189: pembrolizumab or placebo plus pemetrexed and platinum for previously untreated metastatic nonsquamous non-small-cell lung cancer. J Clin Oncol.

[48] Hirano Y, Kaneko H, Konishi T (2022). ASO author reflections: epidural analgesia decreases in-hospital mortality, respiratory complications, and anastomotic leakage after minimally invasive esophagectomy for esophageal cancer. Ann Surg Oncol.

[49] Blom JW, Osanto S, Rosendaal FR (2004). The risk of a venous thrombotic event in lung cancer patients: higher risk for adenocarcinoma than squamous cell carcinoma. J Thromb Haemost.

[50] Akhtar-Danesh G-G, Akhtar-Danesh N, Shargall Y (2022). Venous thromboembolism in surgically treated esophageal cancer patients: a provincial population-based study. TH Open.

[51] Wang H-Y, Wu S-G, Lin Y-T (2022). Risk of thromboembolism in non-small-cell lung cancers patients with different oncogenic drivers, including ROS1, ALK, and EGFR mutations. ESMO Open.

