# Supplementary material for: Thrombotic risk of platinum combination chemotherapy with and without immune checkpoint inhibitors for advanced non-small cell lung cancer: a nationwide inpatient database study
Source: Cancer Immunol Immunother. 2023 Aug 4;72(11):3581–91. doi: 10.1007/s00262-023-03508-1 (PMC10576683; doi:10.1007/s00262-023-03508-1)
Supplement: Supplementary file 1 — Supplementary file1 (DOCX 34 KB) [file 262_2023_3508_MOESM1_ESM.docx]

**Thrombotic risk of platinum combination chemotherapy with and without immune checkpoint inhibitors for advanced non-small cell lung cancer: a nationwide inpatient database study**

**Chikako Iwai, Taisuke Jo, Takaaki Konishi, Asahi Fujita, Nobuaki Michihata, Hiroki Matsui, Kiyohide Fushimi, Hideo Yasunaga**

**Supplemental Table 1** Definitions of ICD-10 Code

**Supplemental Table 2** Details of immune checkpoint inhibitor in each regimen before and after overlap weighting

**Supplemental Table3** Details of the incidence of thromboembolism before and after overlap weighting

**Supplemental Table 4** SHR/HR for outcomes within 6 months after the start of platinum combination chemotherapy stratified by age (<75 and ≥75 years)

**Supplemental Table 5** SHR/HR for outcomes within 6 months after the start of platinum combination chemotherapy stratified by pembrolizumab and atezolizumab regimen

**Supplemental Table 1** Definitions of ICD-10 Codes

| **Diseases** | **ICD-10 codes** |
| --- | --- |
| Lung cancer | C34 |
| Venous thromboembolism | I26.0, I26.9, I80.1, I80.2, I80.3, I80.8, I80.9, I82.8 |
| Arterial thromboembolism | I20.0, I20.1, I20.8, I20.9, I21.0, I21.1, I21.2, I21.3, I21.4, I21.9, I63, G45.9, |
|  | I74.0, I74.1, I74.2, I74.3, I74.4, I74.5, I74.8, I74.9 |
| ***Comorbidities*** |  |
| Atrial fibrillation | I48 |
| Autoimmune disease | M05.0, M05.2, M05.3, M05.8, M05.9, M06, M30, M31, M32, M33, M34, M35, M36 |
| Chronic kidney disease | N18 |
| Chronic pulmonary disease | I27.8, I27.9, J40–J47, J60–J67, J68.4 |
| Congestive heart failure | I09.9, I11.0, I13.0, I13.2, I25.5, I42.0, I42.5–I42.9, I43, I50, P29.0 |
| Chronic interstitial pneumonia | J84.1, J84.8, J84.9 |
| Dementia | F00–F03, F05.1, G30, G31.1 |
| Diabetes with chronic complication | E10.2–E10.5, E10.7, E11.2–E11.5, E11.7, E12.2–E12.5, E12.7, E13.2–E13.5, E13.7, |
|  | E14.2–E14.5, E14.7 |
| Diabetes without chronic complication | E10.0, E10.1, E10.6, E10.8, E10.9, E11.0, E11.1, E11.6, E11.8, E11.9, E12.0, E12.1, |
|  | E12.6, E12.8, E12.9, E13.0, E13.1, E13.6, E13.8, E13.9, E14.0, E14.1, E14.6, E14.8, E14.9 |
| Dyslipidemia | E78 |
| Hemiplegia or paraplegia | G04.1, G11.4, G80.1, G80.2, G81, G82, G83.0–G83.4, G83.9 |
| Hypertension | I10, I11, I12, I13, I14, I15 |
| Intracerebral bleeding | I60, I61, I62 |
| Mild liver disease | B18, K70.0–K70.3, K70.9, K71.3–K71.5, K71.7, K73, K74, K76.0, K76.2–K76.4, |
|  | K76.8, K76.9, Z94.4 |
| Moderate or severe liver disease | I85.0, I85.9, I86.4, I98.2, K70.4, K71.1, K72.1, K72.9, K76.5, K76.6, K76.7 |
| Other interstitial pneumonia | J70.0, J70.1, J70.2, J70.3, J70.4, J99.0, J99.1, M05.1 |
| Peptic ulcer disease | K25–K28 |
| Psychoses | F20, F22, F23, F24, F25, F28, F29, F30.2, F31.2, F31.5 |

ICD-10, International Classification of Diseases, Tenth Revision

**Supplemental Table 2** Details of immune checkpoint inhibitor in each regimen before and after overlap weighting

| **Regimens** | **Added ICI** | **Before overlap weighting** | **After overlap weighting** |
| --- | --- | --- | --- |
| CDDP plus PEM | Pembrolizumab | 882 | 5,090 |
|  | Atezolizumab | 12 | 69 |
| CBDCA plus PEM | Pembrolizumab | 2,974 | 15,841 |
|  | Atezolizumab | 76 | 402 |
| CBDCA plus nabPTX | Pembrolizumab | 2,034 | 10,217 |
|  | Atezolizumab | 185 | 902 |
| CBDCA plus PTX | Pembrolizumab | 296 | 1,798 |
| CBDCA plus PTX plus BEV | Atezolizumab | 718 | 3,584 |
|  | Total | 7,177 | 37,903 |

ICI, immune checkpoint inhibitor; CDDP, cisplatin; CBDCA, carboplatin; PEM, pemetrexed; nabPTX, nab-paclitaxel; PTX, paclitaxel; BEV, bevacizumab

**Supplemental Table3** Details of the incidence of thromboembolism before and after overlap weighting

|  | **Before overlap weighting** | | | |  | **After overlap weighting** | | | |
| --- | --- | --- | --- | --- | --- | --- | --- | --- | --- |
|  | **ICI** | | **non-ICI** | |  | **ICI** | | **non-ICI** | |
|  | **n=7,177** | | **n=68,630** | |  | **n=37,903** | | **n=37,903** | |
| **Venous thromboembolism** | 96 | (1.3) | 665 | (0.97) |  | 505 | (1.3) | 389 | (1.0) |
| **Deep vein thrombosis** | 69 | (0.96) | 487 | (0.71) |  | 361 | (0.95) | 283 | (0.75) |
| **Pulmonary embolism** | 37 | (0.52) | 286 | (0.42) |  | 198 | (0.52) | 164 | (0.43) |
| **Arterial thromboembolism** | 38 | (0.52) | 351 | (0.51) |  | 204 | (0.54) | 221 | (0.58) |
| **Ischemic heart disease** | 9 | (0.13) | 76 | (0.11) |  | 48 | (0.13) | 52 | (0.14) |
| **Ischemic brain disease** | 24 | (0.33) | 206 | (0.30) |  | 132 | (0.35) | 133 | (0.35) |
| **Peripheral arterial embolism** | 5 | (0.07) | 78 | (0.11) |  | 24 | (0.06) | 41 | (0.11) |

ICI, immune checkpoint inhibitor

All outcomes were events requiring hospitalization within 6 months after the start of platinum combination chemotherapy. Data are shown in n (%).

**Supplemental Table 4** SHR/HR for outcomes within 6 months after the start of platinum combination chemotherapy stratified by age (<75 and ≥75 years)

|  | **Age** | **SHR** | **95% CI** | ***P* value** |
| --- | --- | --- | --- | --- |
| **Venous thromboembolism** | <75 | 1.25 | 0.97–1.62 | 0.084 |
|  | ≥75 | 1.45 | 0.83–2.52 | 0.19 |
| **Arterial thromboembolism** | <75 | 0.83 | 0.54–1.29 | 0.42 |
|  | ≥75 | 1.59 | 0.80–3.14 | 0.19 |
|  | **Age** | **HR** | **95% CI** | ***P* value** |
| **All-cause in-hospital death** | <75 | 0.67 | 0.60–0.73 | <0.001 |
|  | ≥75 | 0.75 | 0.62–0.92 | 0.006 |

SHRs, subdistribution hazard ratios; HR, hazard ratio; CI, confidence interval

**Supplemental Table 5** SHR/HR for outcomes within 6 months after the start of platinum combination chemotherapy stratified by pembrolizumab and atezolizumab regimen

|  | **ICI** | **SHR** | **95% CI** | ***P* value** |
| --- | --- | --- | --- | --- |
| **Venous thromboembolism** | Pembrolizumab | 1.29 | 1.01–1.64 | 0.042 |
|  | Atezolizumab | 0.91 | 0.49–1.66 | 0.77 |
| **Arterial thromboembolism** | Pembrolizumab | 1.01 | 0.71–1.46 | 0.94 |
|  | Atezolizumab | 0.39 | 0.11–1.48 | 0.17 |
|  | **ICI** | **HR** | **95% CI** | ***P* value** |
| **All-cause in-hospital death** | Pembrolizumab | 0.68 | 0.63–0.75 | <0.001 |
|  | Atezolizumab | 0.58 | 0.46–0.75 | <0.001 |

ICI, immune checkpoint inhibitor; SHRs, subdistribution hazard ratios; HR, hazard ratio; CI, confidence interval
